# Association of Chest Pain Protocol–Discordant Discharge With Outcomes Among Emergency Department Patients With Modest Elevations of High-Sensitivity Troponin

**DOI:** 10.1001/jamanetworkopen.2022.26809

**Published:** 2022-08-15

**Authors:** Ayesha Khan, Muhammad S. Saleem, Keith D. Willner, Luke Sullivan, Elsie Yu, Osama Mahmoud, Amro Alsaid, Martin E. Matsumura

**Affiliations:** 1 Geisinger Northeast Internal Medicine Residency Program, Wilkes-Barre, Pennsylvania; 2 Department of Emergency Medicine, Geisinger Wyoming Valley Hospital, Wilkes-Barre, Pennsylvania; 3 Heart Institute, Geisinger Medical Center, Danville, Pennsylvania; 4 Geisinger Health System, Laboratory Medicine, Danville, Pennsylvania; 5 Pearsall Heart Hospital, Geisinger Health System, Wilkes Barre, Pennsylvania

## Abstract

**Importance:**

Accelerated diagnostic protocols (ADPs) for chest pain using high-sensitivity troponin (hsTn) levels have excellent sensitivity and negative predictive value for rapid risk stratification of patients with chest pain. However, little is known about the outcomes of patients who are discharged despite abnormal ADP results, ie, after “ruling-in” with a modest elevation of hsTn.

**Objective:**

To determine outcomes of patients discharged following ADP, including those who were ruled in with modestly elevated levels of hsTnT but discharged nonetheless.

**Design, Setting, and Participants:**

This retrospective cohort study included patients with chest pain who presented to the emergency departments (EDs) of a large multisite health system ED between January 2017 to September 2019. Patients were assessed using an ADP, had a peak hsTnT level measured between the limit of quantitation and 52 ng/L, were discharged, and had follow-up in the electronic medical record. Data analysis was conducted from January 2017 to September 2019.

**Exposures:**

Application of an hsTnT ADP.

**Main Outcomes and Measures:**

Thirty-day major adverse cardiac events (MACE), including myocardial infarction, urgent coronary revascularization, and all-cause death, comparing patients who were discharged following ADP-concordant vs ADP-discordant results.

**Results:**

Of 10 342 patients with chest pain (mean [SD] age 51 [17] years; 5902 [57%] women) discharged following ADP, 29 (0.28%) had MACE. Patients with MACE were older (median [IQR] age, 66 [53-75] years vs 50 [38-62] years; *P* < .001) and more likely to have prior CAD (12 [41.4%] vs 1805 [17.5%]; *P* = .002) and hyperlipidemia (13 [44.8%] vs 2248 [21.8%]; *P* = .006). Additionally, patients with MACE were 5-fold more likely to have been discharged despite ADP discordance (16 [55.2%] vs 1145 [11.1%]; *P* < .001). A multivariable logistic regression analysis revealed only ADP discordance was independently associated with MACE (odds ratio, 6.42 [95% CI, 2.94-14.0]; *P* < .001). When stratified by peak hsTnT level, there were no differences in MACE between ADP-concordant and -discordant discharges provided the peak hsTnT measured was less than 12 ng/L. In contrast, patients with peak hsTnT level between 12 and 51 ng/L were significantly more likely to have MACE if they were discharged after ADP-discordant vs -concordant hsTnT series (14 of 609 [2.30%] vs 5 of 1047 [0.48%]; *P* < .002). Notably, a HEART (history, electrocardiogram, age, risk factors, troponin) score of 4 or greater retrospectively identified the most ADP-discordant discharges (13 of 16 [81.3%]) who had MACE.

**Conclusions and Relevance:**

In this cohort study, an hsTnT ADP identified patients who could be discharged from the ED with low 30-day risk of MACE, provided the discharge was based on ADP-concordant “rule-out.” Conversely, the rate of MACE was significantly higher among patients discharged despite ADP discordance. Most patients with ADP-discordant discharges who experienced MACE had a HEART score of 4 or greater, suggesting that application of this score may augment discharge decisions of patients despite ADP-discordant troponin series.

## Key Points

**Question:**

In patients presenting to the emergency department with chest pain, what are the outcomes following discharge despite modest elevations of high-sensitivity troponin?

**Findings:**

In this cohort study of 10 342 patients with chest pain who were discharged following application of a high-sensitivity troponin–based advanced diagnostic protocol (ADP), patients with ADP-discordant discharges had significantly higher 30-day adverse cardiac outcomes vs those with ADP-concordant discharges when the peak troponin T level was 12 to 51 ng/L but not when patients had lower peak values. Most patients with ADP-discordant discharges who experienced major adverse cardiac events had a retrospectively calculated HEART (history, electrocardiogram, age, risk factors, troponin) score of 4 or greater.

**Meaning:**

In this study, physician discharge decisions that deviated from a chest pain ADP were associated with worse outcomes vs a troponin level–only strategy, but addition of a clinical risk score as a predischarge check may have identified most patients who had adverse cardiac events.

## Teaser

This cohort study evaluates outcomes of patients discharged following accelerated diagnostic protocols for chest pain, including patients with modestly elevated levels of high-sensitivity troponin T.

## Introduction

Approximately 7.6 million patients per year in the United States present to the emergency department (ED) with chest pain or other symptoms suggestive of acute myocardial infarction (MI).^1^ Acute MI is a leading cause of disability and death, and methods to efficiently and safely risk stratify patients with chest pain are evolving.^2^ High-sensitivity troponins (hsTns) are well-established in the diagnosis of MI as the cause of chest pain and have facilitated the development of accelerated diagnostic protocols (ADPs) aimed at rapidly ruling in or ruling out MI with acceptable diagnostic accuracy.^3,4,5,6^ Multiple algorithms have been devised to guide triaging patients with chest pain in the ED based on the linear elevation of hsTn level.^5,7^ These ADPs have sensitivities and negative predictive values approaching 100%.^8^ However, patients with chest pain who have hsTn levels between the limit of quantitation (LOQ) and levels unequivocally considered “ruled-in” pose a challenge to decisions regarding discharge. To address this challenge, a number of protocols involve serial measurements of hsTn levels over 1 to 3 hours after presentation, with discharge decisions based on the peak hsTn level and change in hsTn concentration with time.^9^ Several of these protocols have excellent sensitivity and negative predictive value.^10,11^ Conversely, the positive predictive value of these ADPs is comparatively low, and multiple studies have mixed results addressing the value of adding bioclinical risk scores to the ADP to augment the determination of patients eligible for ED discharge.^12^ Despite the low positive predictive value of an abnormal ADP result, the outcomes of patients who are discharged from the ED despite a rule-in ADP result based on a risk score, physician opinion of a patient’s cardiac risk, or other factors are unknown.

In the present study, we assessed the effectiveness of an hsTnT ADP to identify patients with chest pain eligible for ED discharge and compare outcomes of patients who were discharged after an ADP rule-out vs those who were discharged despite an ADP rule-in based on modest elevations in hsTnT level; ie, whether physician decisions to discharge patients who were ruled-in were associated with poor patient outcomes and whether these decisions were similarly discordant with the results of a validated bioclinical risk score.

## Methods

The present study is a retrospective cohort study, for which we followed the Strengthening the Reporting of Observational Studies in Epidemiology (STROBE) reporting guidelines.^13^ The study was reviewed and approved by the Geisinger Health System institutional review board with a waiver of informed consent because the study was retrospective.

**Figure 1.**
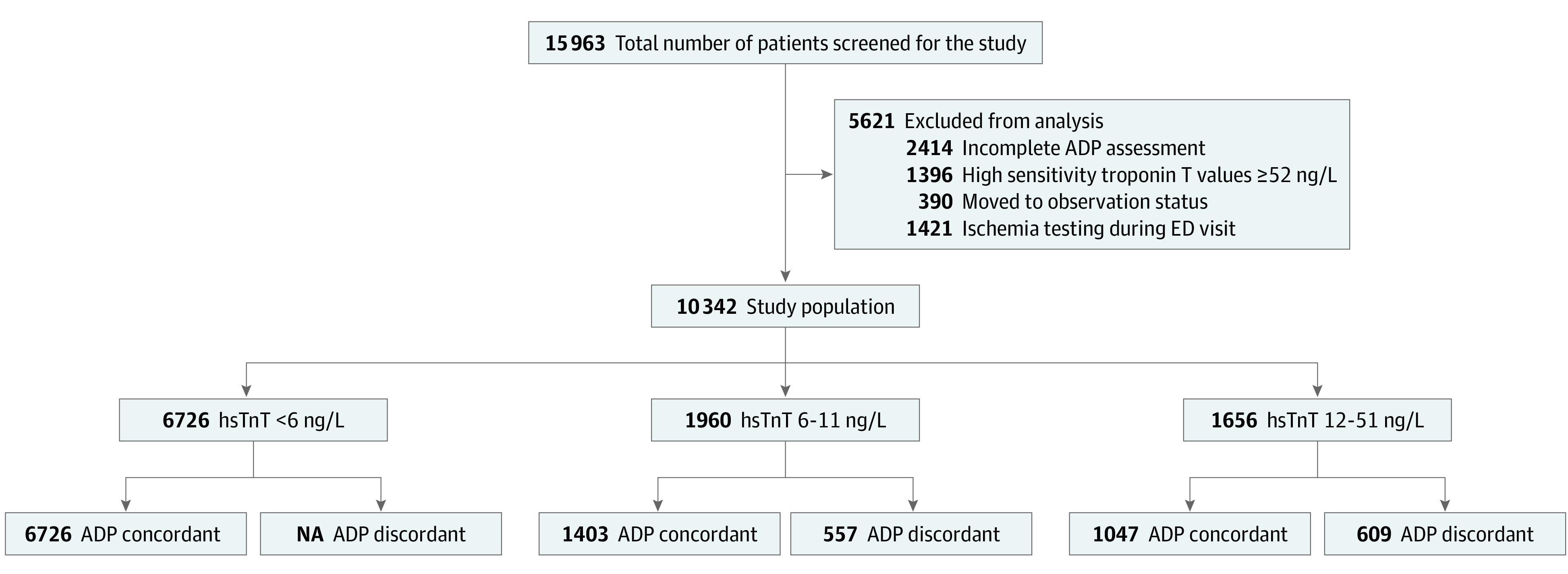
Study Flow Diagram ADP indicates advanced diagnostic protocol; ED, emergency department; hsTnT, high-sensitivity troponin T; NA, not applicable.

Patients with primary or secondary diagnosis of chest pain identified by *International Statistical Classification of Diseases and Related Health Problems, Tenth Revision *(*ICD-10*) codes who were discharged from any of the Geisinger Health System’s acute care hospital EDs following application of an hsTnT-based ADP from January 2017 to September 2019 were included. Geisinger Health System is an integrated hospital system consisting of multiple EDs, and all sites initiated the same ADP throughout the study period. Patients were excluded from the study if they were discharged with an *ICD-10* code for noncardiac sources of chest pain or if they did not have documented follow-up in the electronic medical record following ED discharge (Figure 1). *ICD*-*10* codes used in the data extraction are listed in the eTable in the Supplement.

**Figure 2.**
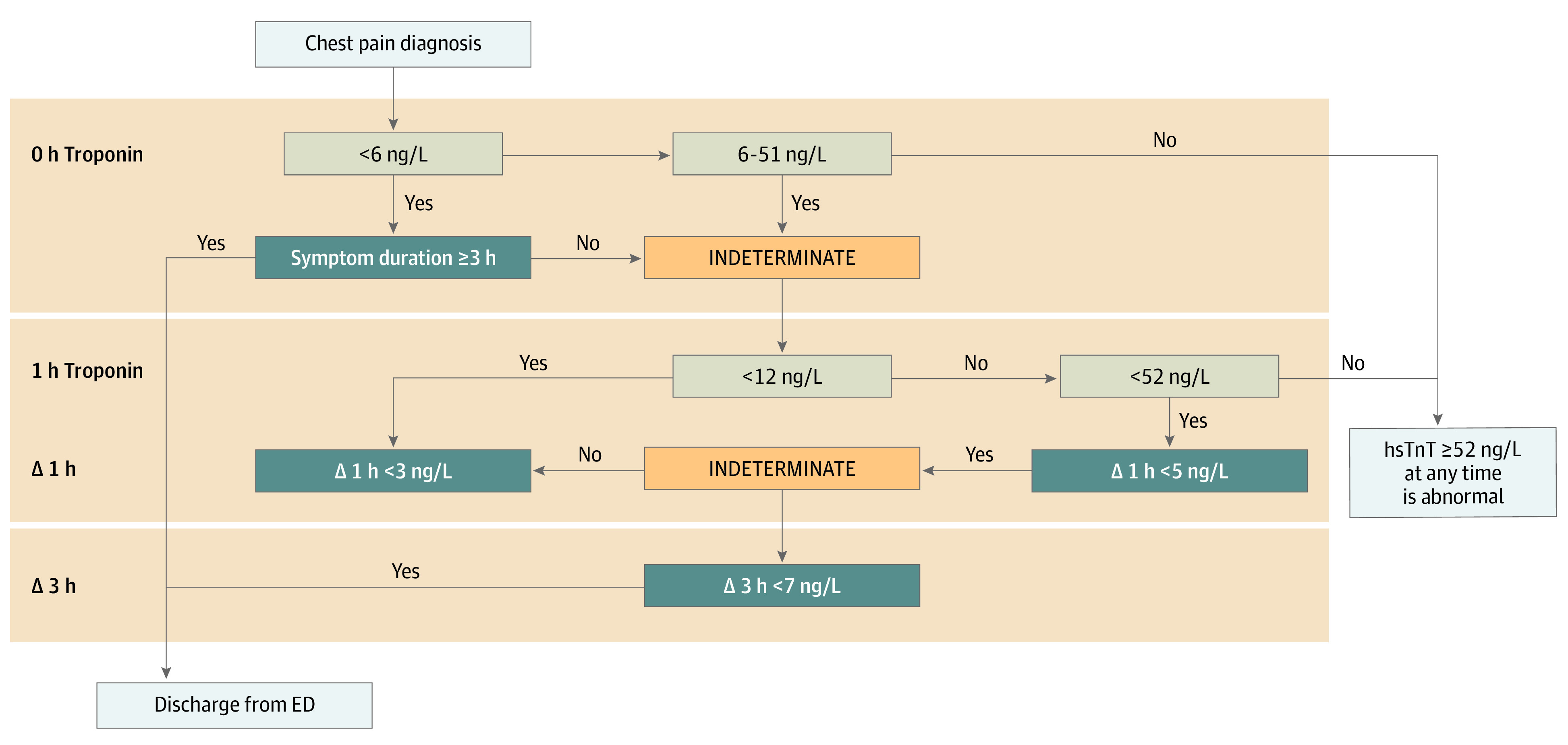
Summary of High-Sensitivity Troponin T (hsTnT) Accelerated Diagnostic Protocol ED indicates emergency department.

The ADP was adapted from consensus guidelines and was a priori published and validated. It used 0-, 1-, and 3-hour hsTnT measurements.^10,11^ The algorithm is summarized in Figure 2. In this ADP, serial testing is indicated for hsTnT determinations between LOQ and 51 ng/L, and levels of 52 ng/L and greater are considered unequivocally positive (to convert hsTnT to micrograms per liter, multiply by 0.001). Decisions to discharge patients were made by the ED clinicians based on the result of the ADP, but admission and discharge decisions were not automated, ie, the clinician’s disposition decision was guided by clinical judgement as well as the outcome of the ADP. Use of the HEART (history, electrocardiogram, age, risk factors, troponin) score to aid in adjudication was recommended but not required as part of the chest pain work-up and discharge decision-making.

Patients were included if they met the following criteria: age 18 years or older, primary diagnosis of chest pain, minimum of 1 measurement of hsTnT with the highest measured value being 51 ng/L or less, and a decision to discharge from the ED without further ischemic testing performed during the index stay. Patients were excluded from this study if they had any hsTnT values of 52 ng/L or greater, as these are considered unequivocally positive by the algorithm. An electronic medical record was used to identify patients meeting study criteria as well as extract data regarding patient demographics and medical history, results of ED evaluation and discharge decision-making, and occurrence of death or myocardial infarction within 30 days of the index ED visit. The HEART score was derived retrospectively via extraction of data in the electronic medical record by the study team.^14^

Levels of hsTnT were measured using the Roche Elecsys Troponin T Gen 5 Immunoassay (Roche Diagnostics). Patients were stratified by the peak measured hsTnT into 3 groups: below the LOQ (<6 ng/L), 6 to 11 ng/L, and 12 to 51 ng/L.^11^ Within these strata, patients were grouped by whether they were discharged with results that met ADP criteria for a rule-out determination. The change in troponin concentration between assays performed in a prespecified time interval was considered abnormal if the absolute change in hsTnT concentration was greater than 3 ng/L for patients with initial values less than 12 ng/L or greater than 5 ng/L for those with initial values of 12 to 51 ng/L. These cutoffs were based on prior data regarding serial changes hsTnT and outcomes and are reflected in subsequent guidelines.^9,15^

We classified an *ADP-discordant* discharge as an ED discharge despite a hsTnT series that met criteria for ruling in by the ADP. The primary end points were major adverse cardiac events (MACE), including MI, urgent revascularization, and all-cause mortality within 30 days following ED discharge. Thirty-day events were determined by data extraction from the electronic medical record, which is a common shared system that was used by all ED and hospital sites included in the analysis. MI was defined as an ED presentation or admission for chest pain accompanied by elevated cardiac biomarkers.^16^ Urgent revascularization was defined as occurrence of acute cardiac symptoms leading to urgent and unplanned ED admission or an urgent outpatient visit leading to hospital admission for cardiac catheterization and percutaneous revascularization or surgical coronary artery bypass grafting.^17^

### Statistical Analysis

Statistical analysis was performed using Sigmastat version 4 (Systat). Data were presented as means and SDs for continuous variables that were normally distributed and medians and 95% CIs for nonnormally distributed data. Categorical data were presented as proportions. Comparisons between data groups were performed using Mann-Whitney *U* tests for nonnormal data and χ^2^ analysis for categorical data. Multivariable logistic regression was performed on independent significant variables. For all comparisons, *P* < .05 was considered significant, and tests were 2-tailed.

## Results

A total of 10 342 patients (mean [SD] age 51 [17] years; 5902 [57%] women) were discharged following application of the ADP (Figure 1). Of these patients, 29 (0.28%) had MACE within 30 days of discharge. Comparison of demographic characteristics and comorbidities of patients with and without 30-day MACE are summarized in Table 1. Patients with MACE vs those without were significantly older median [IQR] age, 66 [53-75] years vs 50 [38-62] years; *P* < .001) and more likely to have a history of CAD (12 [41.4%] vs 1805 [17.5%]; *P* = .002) and hyperlipidemia (13 [44.8%] vs 2248 [21.8%]; *P* = .006). Additionally, patients with MACE were nearly 5-fold more likely to have an ADP-discordant discharge decision (16 [55.2%] vs 1145 [11.1%]; *P* < .001), ie, a discharge despite hsTnT results that ruled-in the patient. In a multivariable regression model including significant univariate factors, only ADP discordance was independently associated with 30-day MACE (OR, 6.42; 95% CI, 2.92-14.03; *P* < .001) (Table 2).

**Table 1.** Demographics and Comorbidities of Patients With vs Without 30-Day MACE

Characteristic	30-d, %		P value
	With MACE (n = 29)	Without MACE (n = 10 313)	
Age, median (IQR)	66 (53-75)	50 (38-62)	<.001
Sex			
Female	13 (44.8)	5889 (57.1)	.25
Male	16 (55.2)	4424 (42.9)	
Prior CAD	12 (41.4)	1805 (17.5)	.002
Diabetes	8 (27.6)	1392 (13.5)	.05
Hypertension	12 (41.4)	2950 (28.6)	.19
Hyperlipidemia	13 (44.8)	2248 (21.8)	.006
Smoker	21 (72.4)	6177 (59.9)	.24
Creatinine level, median (IQR), ng/dL	0.9 (0.7-1.1)	0.8 (0.7-1.0)	.59
ADP discordance	16 (55.2)	1145 (11.1)	<.001

Abbreviations: ADP, accelerated diagnostic protocol; CAD, coronary artery disease; MACE, major adverse cardiac events.

SI conversion Factor: To convert creatinine to micromoles per liter, multiply by 88.4.

**Table 2.** Multivariable Logistic Regression Analysis of Univariate Factors Associated With 30-Day Major Adverse Cardiac Events

Factor	OR (95% CI)	*P* value
Age	1.02 (0.998-1.05)	.08
Prior CAD	1.45 (0.64-3.28)	.37
Hyperlipidemia	1.53 (0.70-3.36)	.29
Protocol-discordant hsTnT level	6.42 (2.94-14.03)	<.001

Abbreviations: CAD, coronary artery disease; hsTnT, high sensitivity troponin T; OR, odds ratio.

Of the 10 342 ED discharges following ADP, 1166 (11.3%) were discharged as ADP discordant, as defined by hsTnT results that indicated a rule-in status. We examined the outcomes in these patients compared with the 9176 patients who were ADP concordant, meaning the results of the ADP indicated rule-out status.

Thirty-day adverse outcomes between ADP-concordant and discordant patients stratified by peak hsTnT are shown in Table 3. Overall, patients with ADP-concordant discharges had significantly lower 30-day MACE vs those with ADP-discordant discharges (13 of 9176 [0.14%] vs 16 of 1166 [1.37%]; *P* < .001). When stratified by peak hsTnT, there remained significant difference between ADP-concordant and discordant patients in the hsTnT range of 12 to 51 ng/L (5 of 1047 [0.48%] vs 14 of 609 [2.30%]; *P* < .002). Conversely, there was no difference between groups with peak hsTnT levels of less than 12 ng/L. It is presumed that ADP-discordant ED discharge decisions were based on clinical factors beyond the ADP result, including clinician and patient risk tolerance and the clinician’s subjective sense of the patient’s risk of MACE. We therefore examined whether objective measures of bioclinical characteristics would have further stratified risk of the ADP-discordant discharges by comparing the HEART score of patients with ADP-discordant ED discharges who had a 30-day MACE vs a randomly selected sample of patients with ADP-discordant discharges who did not have a 30-day MACE. Patients with MACE had a significantly higher median HEART score vs those without MACE (median [IQR], 5 [4-6] vs 3 (2-4]; *P* < .001). Additionally, the percentage of patients with high-risk HEART scores of 4 or greater was significantly higher among patients with ADP-discordant discharges with MACE vs those without MACE (13 of 16 [81.3%] vs 50 of 210 [23.8%], *P* < .001).

**Table 3.** Thirty-Day Major Adverse Cardiac Event Among Patients with ADP-Concordant vs -Discordant Discharges, Overall and Stratified by Peak hsTnT Measurement

Group	Patients, No./total No. (%)			*P* value
	All patients	ADP-concordant discharge	ADP-discordant discharge	
All patients	29/10342 (0.28)	13/9176 (0.14)	16/1166 (1.37)	<.001
Peak hsTnT				
<6 ng/L	3/6726 (0.04)	3/6726 (0.04)	0	NA
6-11 ng/L	7/1960 (0.36)	5/1403 (0.36)	2/557 (0.36)	.68
12-51 ng/L	19/1656 (1.15)	5/1047 (0.48)	14/609 (2.30)	<.002

Abbreviations: ADP, accelerated diagnostic protocol; hsTnT, high sensitivity troponin T; NA, not applicable.

SI conversion factor: To convert hsTnT to micrograms per liter, multiply by 0.001.

## Discussion

A growing body of literature supports the use of hsTn-based ADPs to risk stratify patients who present with chest pain.^5,18^ Accelerated chest pain protocols have consistently demonstrated sensitivity and negative predictive value for MACE in the range 97% to 100%, including ADPs in which decision-making is based on troponin levels only.^19,20^ Conversely, the positive predictive value of ADPs has been shown to be as low as 50% to 60%, confirming that a large number of patients with chest pain who are ruled in with these ADPs will ultimately be found to not have MACE.^20^ Whether physician judgement can identify patients who rule in by hsTn algorithms but are still considered safe to be discharged without further risk stratification is a matter of debate. In the present study, we examined the outcomes associated with a 0-, 1-, and 3-hour ADP, including the rate of MACE associated with discharge from the ED despite ruling-in by the ADP with modest elevations of hsTnT, ie, less than 52 ng/L.^10^ We found the following: (1) in practice, the ADP was associated with very low 30-day MACE; (2) patients with chest pain who were ruled out by the ADP had a low rate of 30-day MACE; (3) when clinician judgement led to ED discharge despite rule-in by ADP (protocol-discordant management), 30-day MACE was significantly higher; and (4) ADP-discordant patients discharged from the ED who had 30-day MACE had significantly higher HEART scores compared with those discordant discharges who did not have MACE, suggesting the addition of the HEART score to an ADP-discordant discharge decision may further risk stratify these patients.

It comes as no surprise that patients who were discharged after ADP-discordant results had higher rates of MACE compared with the extremely low rates of MACE among patients who were ruled out via the ADP. Multiple studies have confirmed the high sensitivity and negative predictive value of ADPs, including hsTn-only algorithms,^20^ but there is little data available on the outcomes of patients who are managed conservatively after ruling-in by ADP. It is notable that in our study MACE was quite low in patients who were discharged after ruling-in when their peak hsTnT was less than 12 ng/L, suggesting that selected patients in this range of hsTnT regardless of serial trends may be reasonably considered for discharge based on clinical suspicion and perhaps after application of additional risk scores.

While ADP discordance was noted in 11.3% of the entire cohort of patients discharged after chest pain ADP application, it is notable that most patients did not have 30-day MACE, consistent with the well-established low positive predictive value of chest pain protocols and the fact that our cohort included patients with chest pain and modest troponin elevations. Retrospective application of the HEART score was reasonably efficacious at identifying those patients with ADP-discordant discharge who ultimately had 30-day MACE, a finding that is intriguing and worth studying further. While a number of studies have evaluated the addition of the HEART score to ADPs among patients with chest pain with variable results, we believe our data are the first to assess the potential prognostic performance of the HEART score to provide a check on patient discharge decisions despite rule-in by an ADP.^4,21,22,23^ Based on our data, a reasonable integration of the HEART score into the ADP could be considered: the HEART score could be a necessary step prior to final disposition of patients who ED clinicians felt were of acceptable risk for discharge despite ADP discordance. In fact, 13 of 16 patients who had 30-day MACE following an ED discharge discordant with the ADP had a HEART score of 4 or greater. If these patients were identified for further ischemic workup and MACE were avoided, the 30-day MACE for patients who had ADP-discordant discharges would be 0.25%, and the 30-day MACE for the entire cohort would be 0.15%, both representing acceptable miss rates. We caution that these data are hypothesis-generating and further work is needed to determine whether the application of a predischarge HEART score can further stratify ruled-in patients into low or high risk and thus identify patients who might be managed as outpatients despite rule-in status. A recent analysis of the the Rapid Assessment of Possible Acute Coronary Syndrome in the Emergency Department with High-Sensitivity Troponin T Study (RAPID-TnT) study found no improvement in classification performance in adding the HEART score to ruled-in patients in terms of identifying 30-day death or MI.^24^ The present study differs from RAPID-TnT in that the HEART score was applied to a cohort of patients with chest pain who ruled-in but whom clinicians felt were candidates for discharge based on factors beyond the ADP result, and this difference may explain the additive value of the HEART score in this study.

### Limitations

The present study has significant limitations that should be noted. First, the study included only those patients who were discharged from the ED after chest pain ADP. Since it did not include patients who were ruled-out via ADP but were admitted or underwent further risk stratification because of physician suspicion of higher risk than that represented by the ADP results, a true assessment of the sensitivity and negative predictive value of the protocol in our center cannot be determined. However, prior studies have demonstrated high sensitivity and negative predictive value of this ADP, and it is therefore unlikely that a significantly large number of patients were overlooked in this series.^19^ Second, the study included only patients who were discharged following ADP and had follow-up documented in the electronic medical record. It is certainly possible that patients who were lost to follow-up after the ADP may have had a different risk profile and significantly different 30-day MACE vs those included in our cohort. Third, the ADP applied in our centers used hsTnT cutoffs based on published guidelines and a validated protocol.^10^ Other ADPs use cutoffs that are similar but not necessarily identical to this protocol, and cutoffs vary by both troponin type and assay manufacturer.^9,11^ This variability should be taken into consideration when applying these data to alternative ADPs.^9,19^ Fourth, the drivers of discharge decisions in the face of ADP discordance in the present study are unknown. Because physician’s subjective sense of patient risk of MACE was not specifically assessed in this study, it may be shortsighted to assume physician decision-making alone drove these decisions. Multiple other factors may have contributed to physician discharge decisions despite ADP discordance, eg, different levels of experience with the hsTnT assay and ADP or differing degrees of risk tolerance. Fifth, as mentioned previously, the HEART score was calculated retrospectively and manually, and thus, the result of the ADP may have biased these calculations. Further validation of the utility of the HEART score in ADP-discordant discharge decisions would benefit from prospective and/or automated calculation of the HEART score.

## Conclusions

The present study supports the utility of an hsTnT-based ADP for identifying patients who are safe for discharge in a large health system. Compared with patients with ADP-concordant discharges, patients who were discharged despite an ADP rule-in had significantly worse 30-day outcomes, owing to increased MACE in those patients and peak hsTnT level greater than 11 ng/L but lower than the unequivocally positive cutoff. Patients who had 30-day MACE and were discharged despite ADP discordance could largely be identified by application of the HEART score. Further study is needed to evaluate the use of the HEART score to check decision appropriateness when discharging patients who are rule-in by an ADP for chest pain.

## Notes

Supplement. **eTable. ***ICD-9* and *ICD-10* Codes for Patient Data Extraction
