# Errors in Figure

**DOI:** 10.1001/jamanetworkopen.2022.35575

**Published:** 2022-09-15

In the Original Investigation titled “Association of Chest Pain Protocol–Discordant Discharge With Outcomes Among Emergency Department Patients With Modest Elevations of High-Sensitivity Troponin,”^1^ published August 15, 2022, there were errors in Figure 2. This article has been corrected.^1^
