# Supplementary material for: Association of Chest Pain Protocol–Discordant Discharge With Outcomes Among Emergency Department Patients With Modest Elevations of High-Sensitivity Troponin
Source: JAMA Netw Open. 2022 Aug 15;5(8):e2226809. doi: 10.1001/jamanetworkopen.2022.26809 (PMC9379744; doi:10.1001/jamanetworkopen.2022.26809)
Supplement: Supplement. — eTable. ICD-9 and ICD-10 Codes for Patient Data Extraction [file jamanetwopen-e2226809-s001.pdf]

Supplemental Online Content

Khan A, Saleem MS, Willner KD, et al. Association of chest pain protocol–discordant discharge with outcomes among emergency department patients with modest elevations of high-sensitivity troponin. *JAMA Netw Open.* 2022;5(8):e2226809. doi:10.1001/jamanetworkopen.2022.26809

eTable. *ICD-9* and *ICD-10* Codes for Patient Data Extraction

This supplemental material has been provided by the authors to give readers additional information about their work.

© 2022 Khan A et al. *JAMA Network Open.*

eTable. *ICD-9* and *ICD-10* Codes for Patient Data Extraction

		ICD 9 Code	ICD 10 Code
ICD code for Inclusion Variables	Chest Pain	786.50, 786.59, 786.51, 413, 413.9	R07.9, R07.89, R07.8, R07.1, I20, I20.0, I 20.8, I20.9
ICD Codes for Exclusion Variables	Acute heart failure	NA	I50.41, I50.9, I50.21, I50.31, I50.23, I50. 33
	Sepsis	NA	A41.9
	Pulmonary Embolism	NA	I26.9
	Pericarditis	NA	I51.4, I30.9

© 2022 Khan A et al. *JAMA Network Open.*
